# High Dietary Phosphorus Is Associated with Increased Breast Cancer Risk in a U.S. Cohort of Middle-Aged Women

**DOI:** 10.3390/nu15173735

**Published:** 2023-08-25

**Authors:** Ronald B. Brown, Philip Bigelow, Joel A. Dubin, John G. Mielke

**Affiliations:** 1 School of Public Health Sciences, University of Waterloo, Waterloo, ON N2L 3G1, Canada; 2 Department of Statistics and Actuarial Science, University of Waterloo, Waterloo, ON N2L 3G1, Canada

**Keywords:** breast cancer, dietary phosphorus, Study of Women’s Health Across the Nation, relative risk, nested case–control study, tumorigenesis, cancer epidemiology

## Abstract

Research has shown that high amounts of dietary phosphorus that are twice the amount of the U.S. dietary reference intake of 700 mg for adults are associated with all-cause mortality, phosphate toxicity, and tumorigenesis. The present nested case–control study measured the relative risk of self-reported breast cancer associated with dietary phosphate intake over 10 annual visits in a cohort of middle-aged U.S. women from the Study of Women’s Health Across the Nation. Analyzing data from food frequency questionnaires, the highest level of daily dietary phosphorus intake, >1800 mg of phosphorus, was approximately equivalent to the dietary phosphorus levels in menus promoted by the United States Department of Agriculture. After adjusting for participants’ energy intake, this level of dietary phosphorus was associated with a 2.3-fold increased risk of breast cancer incidence compared to the reference dietary phosphorus level of 800 to 1000 mg, which is based on recommendations from the U.S. National Kidney Foundation, (RR: 2.30, 95% CI: 0.94–5.61, *p* = 0.07). Despite the lack of statistical significance, likely due to the small sample size of the cohort, the present nested case–control study’s clinically significant effect size, dose–response, temporality, specificity, biological plausibility, consistency, coherence, and analogy with other research findings meet the criteria for inferred causality in observational studies, warranting further investigations. Furthermore, these findings suggest that a low-phosphate diet should be tested on patients with breast cancer.

## 1. Introduction

As global populations increasingly transition to the risk factor profile of Western nations, “dramatic changes in lifestyle” are affecting the prevalence of risk factors for breast cancer and other cancers [1]. For example, a recent meta-analysis found that the highest dietary intake of a Western dietary pattern, including red or processed meats, high-fat dairy products, potatoes, and sweets, was associated with a 14% increased risk of breast cancer compared to the lowest intake [2]. The same study found that the highest intake of a “prudent” dietary pattern, containing fruits and vegetables, fish, whole grains, and low-fat dairy, was associated with an 18% reduced risk of breast cancer compared to the lowest intake. Of relevance, the plant-based foods that predominate in a prudent dietary pattern tend to be lower in the essential mineral phosphorus than the animal-based foods typically found in a Western dietary pattern [3].

Inorganic phosphate (Pi) metabolism is regulated in the body with a sensitive network of endocrine hormones released by the kidney–bone–parathyroid–intestine axis [4]. The accumulation of excess Pi in the tissues of the body due to dysregulated phosphate metabolism can produce a condition known as phosphate toxicity, and evidence supports the association of phosphate toxicity with tumorigenesis [5]. For example, animal studies have shown that excessive dietary phosphate increases cell signaling in the promotion of cancer cell growth [6,7]. Notably, a “regulation-based model” of cancer research proposed by Schipper et al., in *The Lancet* in 1996 [8], suggests that cancer is a disease of dysregulated metabolism and may be reversible. In general, metabolomics is currently contributing to the discovery of important metabolic alterations in the growth of cancer cells, with potential applications for clinical oncology [9].

Phosphorus in the form of dietary phosphate is plentiful in the dietary pattern eaten by contemporary Western populations, including in Canada and the United States [10]. Phosphate intake is also rising as people increase their consumption of foods processed with phosphate additives [11]. Dietary sources contributing the greatest amount of phosphorus in the food Americans eat are milk and dairy products (cheese, ice cream, and yogurt), bakery products (bread, rolls, and tortillas), vegetables (starchy), chicken, “Mexican dishes” (nachos, burritos, and tacos), and pizza [12]. Gastrointestinal bioavailability of phosphorus also varies in different dietary sources. For example, phosphorus in meat and dairy has a higher absorption rate (40–60%) compared to phosphorus bound to phytate in whole grains (20–50%), while phosphate additives widely used by the food industry in ultra-processed food have 90–100% bioavailability [13]. Relatedly, recent systematic reviews and meta-analyses found an increased risk of breast cancer and other cancers associated with increased intake of ultra-processed food [14,15]. Another study found an increased risk of mortality from ovarian cancer and breast cancer associated with ultra-processed food intake [16].

As the intake of nutrients like phosphorus rises above optimal levels for health, the increased concentration may eventually become toxic and even result in death [17]. Although the U.S. dietary reference intake (DRI) for phosphorus is 700 mg/day in adult women and men [18], the 2015–2016 National Health and Nutrition Examination Survey (NHANES) reported that women, on average, consume 1189 mg, and men consume 1596 mg of dietary phosphorus/day [19]. By comparison, guidelines from the U.S. National Kidney Foundation’s Kidney Disease Outcomes Quality Initiative (K/DOQI) recommend that patients with progressive kidney disease restrict phosphorus intake to 800–1000 mg/day, depending on protein requirements [20]. Indeed, higher dietary phosphorus intake starting at about 1400 mg per day has been associated with increased all-cause mortality in the U.S. population [21]. Furthermore, based on Dietary Guidelines for Americans, 2020–2025, published by the United States Department of Agriculture (USDA)*,* a MyPlate 2000-calorie daily menu that includes whole grains and fat-free milk provides approximately 1800 mg phosphorous [22], well above the 1400 mg of dietary phosphorus associated with increased mortality risk [21].

Three cups of fat-free milk, as recommended by USDA menu plans, supplies more than 700 mg of phosphorus, which is sufficient to meet adult requirements but provides only about 13% of calories in a 2000-calorie diet [23]; as a result, overall phosphorus intake would quite reasonably be expected to be even higher when other foods are included. Furthermore, a recent study funded by the U.S. National Cancer Institute found a 50% increased risk of breast cancer incidence associated with the highest milk intake compared to the lowest milk intake [24], possibly related to milk’s high phosphorus content. Three cups of milk a day was also associated with a 44% increased risk of cancer mortality compared to one cup [25]. Also, a systematic review and meta-analysis found that dietary acid load is associated with a 58% increased relative risk of cancer [26], and dietary acid load and phosphorus intake were lower in participants in a randomized controlled trial who consumed a vegan diet compared to a meat-rich diet [27].

The purpose of the present study is to investigate associations of breast cancer incidence with discrete categories of dietary phosphate levels. Phosphate levels are based on dietary guidelines from U.S. health organizations and government agencies, and categories also include levels of phosphate associated with disease in the research literature. The hypothesis in the present study is that the relative risk of breast cancer incidence is more strongly associated with high levels of dietary phosphate compared to low levels of phosphate. The rationale for selecting the National Kidney Foundation (NKF) guidelines for dietary phosphate intake as the reference level in this study is based on numerous findings implicating chronic kidney disease as a risk factor for cancer, such as Lees et al. [28], Wong et al. [29], Stengel [30], Tendulkar et al. [31], Kitchlu et al. [32], Hu et al. [33], Movahhed et al. [34], Wei et al. [35], Guo et al. [36], Na et al. [37], and Yu et al. [38]. Additionally, high serum phosphate levels associated with tumorigenesis [5] are also prevalent in chronic kidney disease [39,40]. Conceivably, a low dietary level of phosphate that is least harmful in chronic kidney disease may also reduce the risk of cancer. Therefore, the hypothesis of the study posits that the lowest level of phosphate intake, represented by the NKF recommendations (800–1000 mg), will reduce the relative risk of breast cancer in the cohort compared to higher dietary phosphate levels.

## 2. Materials and Methods

**Figure 1:**
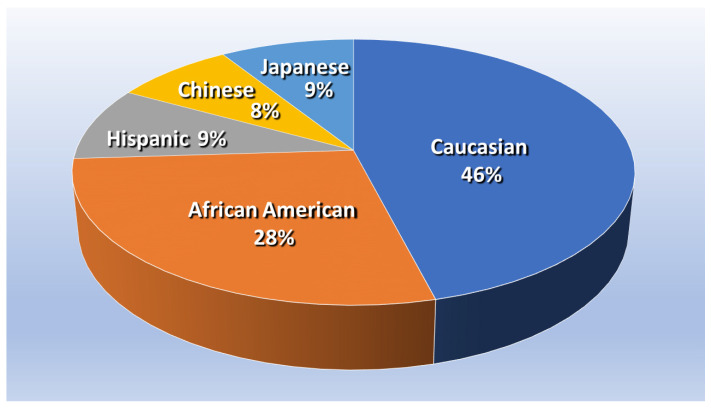
Proportion of SWAN participants, based on About SWAN—Study of Women’s Health Across the Nation, swanstudy.org (accessed on 2 July 2023) [44].

The present study used a nested case–control design to conduct a secondary analysis of cohort data from the Study of Women’s Health Across the Nation (SWAN) [41]. SWAN is funded by the U.S. National Institutes of Health, the National Institute on Aging, the National Institute of Nursing Research, the National Center for Complementary and Alternative Medicine, and the Office of Research on Women’s Health, and the open access dataset for the SWAN study, along with demographic information of the cohort, is freely available online at the study website [42]. SWAN study participants included 3302 multi-ethnic middle-aged American women from a multi-site longitudinal sample. “At the time of enrollment, women were premenopausal, not taking hormones and between 42–52 years of age.” Figure 1 shows the proportion of participants who identified themselves as African American, Caucasian, Chinese, Hispanic, or Japanese [43].

Publicly available data from SWAN used in the present study were collected from baseline interviews and examinations of physical, psychological, biological, and social factors, followed up with 10 annual visits (1997–2007). Food frequency questionnaires (FFQs) were administered to collect dietary data at baseline and at visits 5 and 9. In the present study, each of the 74 breast cancer cases, who self-reported breast cancer during annual follow-up visits, were matched with four controls randomly selected from the cohort, totaling 296 controls consisting of women with similar ages (42–52 years) who were followed over 10 annual assessments. Four controls per case is recommended to increase statistical power in a case–control study, with beyond four matched controls generally leading to negligible increases in power [45]. A list of random numbers was generated using Microsoft Excel to select controls.

### 2.1. Statistical Analysis

Although an odds ratio is most often used in case–control studies to measure the ratio of disease prevalence between exposed and unexposed groups, the present case–control study is nested within a cohort and measures disease incidence or an incidence rate ratio between exposed and unexposed groups, which is represented in the present paper as a risk ratio [46].

“The numerator of an incidence proportion or rate consists only of persons whose illness began during the specified interval. The numerator for prevalence includes all persons ill from a specified cause during the specified interval regardless of when the illness began” [47].

Additionally, odds ratios in cohort studies overestimate the risk ratio [48]. Relative risk formulas with 95% confidence intervals and *p*-values were calculated to four decimal places using online MedCalc Software Ltd. [49]. Statistical significance was set at *p* < 0.05.

### 2.2. Dietary Assessment

Data of dietary phosphorus intake collected at baseline from food frequency questionnaires (FFQ) were cumulatively averaged with FFQ data collected in visits 5 and 9 according to the cumulative average method used by Wallace et al. [50]. Specifically, the sum of phosphorus from three prior FFQ measures over 10 visits was divided by 3 to provide the final cumulative average. Willett stated, “The use of cumulative average measurements (i.e., the average of all measurements for an individual up to the start of each follow-up interval) takes advantage of all prior data and thus should provide a statistically more powerful test of association with cumulative exposure” [17]. For example, a recent study on dietary flavonoids “used the cumulative average intake of flavonoids and other nutrients calculated by averaging their intake at baseline and each follow-up survey” [51]. The same method to calculate cumulative average was used for calorie intake. Also, Wallace et al. handled missing FFQ data for visits 5 and 9 by imputing previously reported values, which is a single-imputation method known as last observation carry-forward (LOCF) [52]. However, noting LOCF can have problems both with biased estimation and artificial reduction in variance [53], missing data in the present study were handled with procedures for multiple imputation calculated with SAS PROC MI using the Fully Conditional Specification Method (FCS).

Additionally, the adjustment method from the *Dietary Assessment Primer* of the National Cancer Institute (NCI) [54] was used in the present study to standardize self-reported dietary information by adjusting for energy intake. According to the NCI, the purpose of energy adjustment is to mitigate “the effects of measurement error in data collected using self-reported dietary assessment instruments.” Energy adjustment is based on “the assumption that individuals tend to misreport intakes of most reported foods and beverages to a similar degree and in the same direction” (e.g., less healthy foods are often underreported more than healthy foods). Information biases from underreported calorie and phosphorus intakes in FFQs were adjusted by estimating each participant’s caloric density of phosphorus, calculated by dividing milligrams of phosphorus intake by caloric intake. This nutrient density quotient was then multiplied by 2000 calories needed for average bodyweight maintenance in women.

To analyze breast cancer risk ratios, energy-standardized dietary phosphorus intakes of participants were grouped into six discrete categories, each spanning 200 mg of phosphorus (P), with 800 to 1000 mg of P as the reference category to which the other five categories were compared. As mentioned in the introduction, the reference category is based on NKF guidelines for P dietary intake [20]. The second phosphate category covers the range from >1000 to 1200 mg of P. The third category ranges from >1200 mg to 1400 mg, which is the level associated with increasing all-cause mortality [21]. The fourth and fifth categories range from >1400 mg to 1600 mg and >1600 to 1800, respectively, and the sixth category, >1800 mg of P, is the approximate level of phosphate in menus recommended by the USDA. Supporting data for categorization and multiple imputation of breast cancer cases and controls is available in the Supplementary Materials.

## 3. Results

Table 1 shows the mean, standard deviation, minimum, and maximum values for P mg intake in the unadjusted and standardized case and control groups, rounded to multiples of 10. Mean unadjusted dietary P levels for the case and control groups are 1120 mg and 1150 mg, respectively, which are approximately equal to the average dietary P intake of 1189 mg reported for U.S. women in the 2015–2016 National Health and Nutrition Examination Survey (NHANES) [55]. Group mean standardized P levels for the case and control groups increased to 1390 mg and 1320 mg, respectively, with an approximate 5% higher mean in the case group compared to the control group.

Interestingly, nine cases initially reported unadjusted dietary P levels below 800 mg (substantially below the NHANES average of 1189 mg of P for U.S. women), which was reduced to two cases <800 mg of P after standardization. Among controls, 62 women initially reported unadjusted dietary P levels below 800 mg which was reduced to eight controls after standardization. Standardization appeared to reduce the proportion of initially reported dietary P levels <800 mg more so in cases (2 out of 9 or 22.2%) than in controls (8 out of 62 or 12.9%). Table 1 also shows that the maximum standardized dietary P intake levels in the cases and controls are 2180 mg and 2450 mg, respectively, which are well below the 4000 mg tolerable upper intake limit (UL) for P to prevent harmful effects according to the Institute of Medicine (IOM) [18]. The IOM notes that the UL was established to guide the use of dietary supplements, and P is not often consumed in supplements in the U.S.

The standardized mean P intakes for cases and controls in our study (1390 and 1320 mg, respectively) are below P levels in MyPlate recommendations (~1800). This suggests that MyPlate recommendations may not be attainable for many people. Additionally, Table 1 shows that minimum levels of standardized dietary P in cases is 770 mg, which meets the daily recommended dietary allowance (RDA) of 700 mg for adult men and women according to the IOM. The IOM also noted that RDAs provide additional P in women for lactation and pregnancy. By contrast, Table 1 shows that the standardized minimum level of dietary P is lower at 570 mg in the controls, yet this level is very close to the IOM’s estimated average requirement (EAR) of 580 mg of P for adult men and women.

Table 2 shows the division of standardized dietary P levels into six discrete dietary intake categories, each spanning 200 mg of P. Estimated relative risks (RR) of breast cancer are calculated by comparing risks from each of five categories of P intake to the reference P intake level of 800–1000 mg (the control level). Risk and RRs in Table 2 are shown rounded to two decimal places, and 95% confidence intervals (CIs) cross the null value of 1, indicating statistical non-significance. However, an increasing risk of breast cancer is associated with exposure to higher P intake levels. Furthermore, the highest level of >1800 mg of P is associated with the highest RR of breast cancer, 2.30, although the *p*-value is non-significant at 0.07.

**Figure 2:**
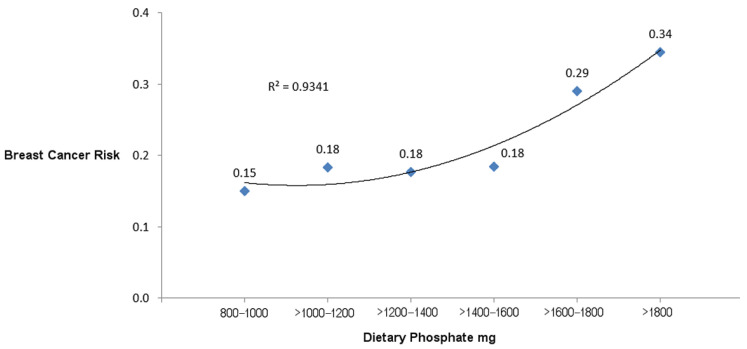
Risks of breast cancer incidence associated with categories of dietary P.

Figure 2 is a graph plotting the risks of breast cancer incidence associated with each category of dietary P mg. A curvilinear regression line fitted to the graph has an R^2^ of 0.9341, indicating a strong correlation between increasing dietary P levels and breast cancer risks.

## 4. Discussion

To the best of the authors’ knowledge, the present study is the first to report an increased risk of self-reported breast cancer incidence associated with a high dietary P intake. Compared to the lowest P intake level in this nested case–control study from the SWAN cohort of middle-aged females, exposure to the highest P intake of >1800 mg is associated with a 2.30 relative risk of breast cancer incidence, although this effect is not statistically significant (95% CI 0.94–5.61, *p* = 0.07). A curvilinear regression line of risks for breast cancer incidence shows a strong correlation with exposure to higher levels of dietary P; R^2^ equals 0.9341.

Additionally, the risk ratios in the study did not reach statistical significance, but this may be due to the study’s limited statistical power and small sample size—breast cancer cases were reported in only 2.2% of the cohort. Nevertheless, the practical significance of the study’s large effect size is important, as “the effect size is the main finding of a quantitative study” [56].

Of particular concern is the 2.30 increased risk of breast cancer incidence associated with the highest level of >1800 mg of P compared to the reference level of 800–1000 mg of P. This high level of P is the approximate amount in menu plans promoted by the USDA. Powerful U.S. government agencies, like the USDA and the U.S. Department of Health and Human Services, currently write the Dietary Guidelines for Americans, “separating the science from the actual guidelines and making the process more political” [57]. Findings of the present study should alert the public to prioritize breast cancer prevention through promotion of dietary recommendations with lower P levels, which might also help reduce the global burden of 3 million new breast cancer cases predicted by 2040 [58].

Although RRs based on uncontrolled observational studies without randomization cannot prove causality, findings of the present study meet the criteria proposed by Bradford Hill, which infer causality in observational studies [59]. The criteria are detailed as follows:

1. Strength of association: The magnitude of the relative risk of breast cancer incidence associated with high dietary P levels is up to 2.3 times greater than associations with low phosphorus levels. “As a measure of effect size, an RR value is generally considered clinically significant if it is less than 0.50 or more than 2.00; that is, if the risk is at least halved, or more than doubled” [60]. A recent review from the International Agency for Research on Cancer (IARC) found that most studies linking various cancers to occupational exposures known to be carcinogenic in humans reported relative risk values well below the 2.30 relative risk in the present study, and approximately one-third of the confidence intervals in the IARC review were not statistically significant [61];
2. Consistency: the association of high dietary P with breast cancer [62] and with other cancers is similar across multiple studies [5];
3. Specificity: the present study shows that P is a specific dietary factor in the association with breast cancer; notably, this does not preclude other risk factors that are associated with breast cancer;
4. Temporality: exposure to high dietary P precedes breast cancer incidence, as revealed in the present nested case–control study’s longitudinal data;
5. Biological gradient: compared to the lowest level of P intake, increasing levels of dietary P in the present study are associated with increasing risk of breast cancer;
6. Plausibility: higher dietary P levels are associated with dysregulated phosphate metabolism and phosphate toxicity, which may lead to tumorigenesis [5,62,63,64,65];
7. Coherence: dysregulated phosphate metabolism and phosphate toxicity fit the regulation-based model of cancer, which proposes that cancer is caused by dysregulated metabolic factors [8];
8. Experimental evidence: Laboratory animal experiments confirm an association between high dietary P feeding and tumorigenesis [6,7]. Importantly, P from dietary sources in these animal experiments are not administered at the maximum tolerated dosages for chemical agents, which are often used in carcinogenic studies [66];
9. Analogy: overgrowth of algae blooms in eutrophication, caused by excessive phosphate fertilizer agricultural runoff [67], is analogous to the ecosystem dynamics of cancer cell overgrowth [68] associated with high dietary P [2,10].

The study’s main strength is that it is the first report to show a large positive dose-dependent association between self-reported breast cancer incidence and increasing levels of dietary P intake in a cohort of middle-aged U.S. women. Limitations of the study include the small sample size of 3302 women in the SWAN cohort compared to nationwide studies of over 161,000 women in the Women’s Health Initiative [69] and 280,000 women in the Nurses’ Health Study [70]. However, the SWAN cohort provides the advantage of a broad ethnic cross-section of middle-aged women in the national population. Furthermore, Pink SWAN, supported by the National Cancer Institute, doubled the follow-up period of the SWAN cohort from 10 to 20 years, and Avis et al. identified 152 breast cancer cases [71], which is approximately twice the sample size of the present study.

Additionally, the nested case–control design of this study has certain limitations common to observational studies:

“The major disadvantage of nested case–control studies is that not all pertinent risk factors are likely to have been recorded. Furthermore, because many different healthcare professionals will be involved in patient care, risk factors and outcome(s) will probably not have been measured with the same accuracy and consistency throughout. It may also be problematic if the diagnosis of the disease or outcome changes with time”.

[72]

Nevertheless, among epidemiological observational studies, the nested case–control design ranks high, providing the advantage of observing disease incidence within a cohort [73].

Other study limitations include the reliance on cohort participants to self-report breast cancer incidence, which may be prone to inaccuracies and information bias, unless credible proof of diagnosis is presented to verify the diagnosis. Study limitations also include standardization of self-reported dietary intake from FFQ data. Although standardization is intended to provide a more realistic estimation of dietary intake to improve validity of the study, standardization cannot estimate actual dietary intake levels, and adjustments are based on averages rather than individual caloric needs of women. More accurate dietary information can be obtained using intervention studies with controlled feeding of participants—which can be very expensive. Furthermore, researchers have found a correlation between dietary phosphate intake and phosphate excreted in 24 h urine collection, which has potential use as a biomarker to estimate dietary phosphate intake in clinical studies [74]. However, compared to short-term measures such as a 24 h recall, FFQs are the most often used dietary tool for epidemiological studies with long follow-up periods [75].

Finally, potential confounding factors were not controlled in this observational study, such as exposures to environmental carcinogens, including alcohol and tobacco, and other risk factors like obesity, low physical activity, and family history of breast cancer [76]. Phosphorus needs may also decline during menopause compared to the reproductive years—which could explain findings of a study in which women at post menopause had increasing levels of serum P [77], which could be related to increasing breast cancer risk as women age [78]. Additionally, a participant’s individual renal function may modify the regulatory effect of dietary phosphate on breast cancer. Future studies should control for effect modification by stratifying the results according to the participant’s estimated glomerular filtration rate or other biomarkers of renal function. Furthermore, renal function declines with age [79], and so, findings of dietary phosphate and breast cancer in this middle-aged female SWAN cohort cannot be generalized to other segments of the population.

For future research on cancer therapies, Kuang et al. wrote, “our simulation results show that if an artificial mechanism (treatment) can cut the phosphorus uptake of tumor cells in half, then it may lead to a three-quarter reduction in ultimate tumor size, indicating an excellent potential of such a treatment” [63]. Furthermore, according to the National Institute of Cancer of the U.S. National Institutes of Health, “When evidence emerges from an epidemiologic study that a dietary component is associated with a reduced risk of cancer, a randomized trial may be done to test this possibility” [80]. Based on the epidemiologic evidence in the present study finding a clinically significant reduced risk of breast cancer incidence associated with low levels of dietary P compared to higher levels, further clinical studies are warranted to test a low-phosphate diet on tumor reduction in breast cancer patients.

## 5. Conclusions

Risk factors for breast cancer include the Western diet, which is high in the essential mineral P. Research has shown that higher amounts of dietary P are associated with disease and mortality. The present nested case–control study measured risk ratios of dietary P levels associated with self-reported breast cancer in middle-aged women from the SWAN cohort. Results in ten annual follow-up visits found that the highest dietary intake of P was associated with a clinically significant 2.30 relative risk of breast cancer incidence compared to the lowest intake level recommended by the U.S. NKF to treat chronic kidney disease. The highest level of P intake is within the approximate range promoted by the USDA. Evidence supports the criteria to infer breast cancer causation from high dietary P intake in observational studies, and further studies with larger cohorts are warranted. Additionally, clinical and preclinical studies with breast cancer patients should test the effect of a low-phosphate diet already in use for patients with chronic kidney disease.

## Figures and Tables

**Table 1:** P mg intake in case and control groups.

Group	Mean	SD	Min.	Max.
Cases, unadjusted (*N* = 74)	1120	330	360	1850
Cases, standardized	1390	340	770	2180
Controls, unadjusted (*N* = 296)	1150	380	330	2620
Controls, standardized	1320	290	570	2450

**Table 2:** Relative risks of breast cancer cases associated with dietary P levels compared to reference level of 800–1000 mg of P.

Dietary P	Breast Cancer Cases	Controls	Risk	Relative Risk
800–1000 mg P	6	34	0.15	Reference
>1000–1200 mg P	13	58	0.18	1.22 (95% CI 0.50–2.96) *p* = 0.66
>1200–1400 mg P	20	93	0.18	1.18 (95% CI 0.51–2.72) *p* = 0.70
>1400–1600 mg P	14	62	0.18	1.23 (95% CI 0.51–2.95) *p* = 0.65
>1600–1800 mg P	9	22	0.29	1.94 (95% CI 0.77–4.86) *p* = 0.16
>1800 mg P	10	19	0.34	2.30 (95% CI 0.94–5.61) *p* = 0.07

## Data Availability

https://www.swanstudy.org/swan-research/data-access/ (accessed on 2 July 2023).

## Supplementary Materials

The following supporting information can be downloaded at https://www.mdpi.com/article/10.3390/nu15173735/s1, Categorized Breast Cancer Cases.xlsx; Categorized Random Controls.xlsx; Multiple Imputed Breast Cancer Cases.rtf; Multiple Imputed Random Controls.rtf.
