# Supplementary material for: High Dietary Phosphorus Is Associated with Increased Breast Cancer Risk in a U.S. Cohort of Middle-Aged Women
Source: Nutrients. 2023 Aug 25;15(17):3735. doi: 10.3390/nu15173735 (PMC10490459; doi:10.3390/nu15173735)
Supplement: Supplementary file 1 [file nutrients-15-03735-s001.zip › Multiple Imputed Breast Cancer Cases.rtf]

Model Information	
Data Set	WORK.IMPORT	
Method	FCS	
Number of Imputations	25	
Number of Burn-in Iterations	20	
Seed for random number generator	962454001	


FCS Model Specification	
Method	Imputed Variables	
Regression	DTTKCAL0 DTTKCAL5 DTTKCAL9 DTTPHOS0 DTTPHOS5 DTTPHOS9	


Missing Data Patterns	
Group	DTTKCAL0	DTTKCAL5	DTTKCAL9	DTTPHOS0	DTTPHOS5	DTTPHOS9	Freq	Percent	
									
1	X	X	X	X	X	X	43	58.11	
2	X	X	.	X	X	.	9	12.16	
3	X	.	X	X	.	X	2	2.70	
4	X	.	X	X	.	.	1	1.35	
5	X	.	.	X	.	.	15	20.27	
6	.	X	X	.	X	X	2	2.70	
7	.	.	X	.	.	X	2	2.70	

Missing Data Patterns	
Group	Group Means	
	DTTKCAL0	DTTKCAL5	DTTKCAL9	DTTPHOS0	DTTPHOS5	DTTPHOS9	
1	1822.341860	1737.145581	1577.644419	1138.855349	1217.360930	1076.758372	
2	1854.398889	1306.041111	.	1085.432222	944.501111	.	
3	1308.700000	.	1238.395000	1152.165000	.	1407.930000	
4	2169.640000	.	1299.270000	1158.040000	.	.	
5	1604.994000	.	.	974.232000	.	.	
6	.	2457.070000	1743.975000	.	1760.880000	1416.045000	
7	.	.	1084.955000	.	.	560.705000	


Variance Information (25 Imputations)	
Variable	Variance	DF	Relative
Increase
in Variance	Fraction
Missing
Information	Relative
Efficiency	
	Between	Within	Total					
DTTKCAL0	264.857673	6349.830974	6625.282954	67.791	0.043379	0.041714	0.998334	
DTTKCAL5	1952.626406	4138.599298	6169.330760	39.236	0.490681	0.335142	0.986772	
DTTKCAL9	758.234125	3725.349359	4513.912849	54.59	0.211675	0.176787	0.992978	
DTTPHOS0	112.656143	1875.018498	1992.180887	66.26	0.062486	0.059082	0.997642	
DTTPHOS5	849.678692	2517.901744	3401.567585	45.833	0.350953	0.263910	0.989554	
DTTPHOS9	419.312519	2586.506723	3022.591743	57.776	0.168600	0.145756	0.994204	


Parameter Estimates (25 Imputations)	
Variable	Mean	Std Error	95% Confidence Limits	DF	Minimum	Maximum	Mu0	
DTTKCAL0	1767.901608	81.395841	1605.470	1930.334	67.791	1724.472630	1797.882354	0	
DTTKCAL5	1666.381128	78.545087	1507.539	1825.223	39.236	1583.191658	1748.840256	0	
DTTKCAL9	1496.121994	67.185660	1361.456	1630.788	54.59	1432.774450	1548.109698	0	
DTTPHOS0	1100.324657	44.633854	1011.217	1189.432	66.26	1082.523330	1128.639388	0	
DTTPHOS5	1181.157626	58.322959	1063.748	1298.567	45.833	1121.952021	1230.814391	0	
DTTPHOS9	1043.390509	54.978102	933.331	1153.450	57.776	996.414219	1073.363483	0	

Parameter Estimates (25 Imputations)	
Variable	t for H0:
Mean=Mu0	Pr > |t|	
DTTKCAL0	21.72	<.0001	
DTTKCAL5	21.22	<.0001	
DTTKCAL9	22.27	<.0001	
DTTPHOS0	24.65	<.0001	
DTTPHOS5	20.25	<.0001	
DTTPHOS9	18.98	<.0001	

Obs	_Impu
tation_	BRST
CAN	SWANID	DTTKCAL0	DTTKCAL5	DTTKCAL9	DTTPHOS0	DTTPHOS5	DTTPHOS9	
1	1	1	11211	793.76	869.05328804	1014.13	631.35	904.87086385	993.97	
2	1	1	13122	2072.34	2919.98	2117.39	1168.75	1579.24	979.1	
3	1	1	15691	2177.36	1114.36	477.1538909	1204.03	522.4	-21.59186432	
4	1	1	16265	2011.46	1678.5603418	1441.3395566	1680.81	1849.6259127	1320.4354766	
5	1	1	19889	970.12	1378.06	1358.62	516.09	716.81	817.22	
6	1	1	20095	1304.99	2031.81	746.77	656.75	1238.74	594.54	
7	1	1	20745	967.17	1318.63	944.01	813.16	797.85	821.61	
8	1	1	22323	2169.64	1295.400277	1299.27	1158.04	909.07720839	1286.9068799	
9	1	1	22878	1564.18	1193.14	1297.43	882.5	894.56	814.4	
10	1	1	22886	1388.79	1988.7907594	1936.690639	602.07	1740.7028635	1645.9112387	
11	1	1	24089	2108.7596276	1491.59	2323.65	1306.143618	1539.09	1958.76	
12	1	1	25038	2289.1	1966.88	1468.37	1822.6	1148.44	900.85	
13	1	1	25175	2936.61	1013.42	2221.37	1378.9	473.39	784.9	
14	1	1	27347	1503.8	1473.25	1234.18	1293.52	1220.43	568.43	
15	1	1	27473	1965.97	1823.05	1529.76	1377	1327.7	838.19	
16	1	1	28364	1443.09	1313.54	1003.29	717.48	859.15	992.44	
17	1	1	29481	2085.21	2045.26	2470.56	1684.38	1804.95	1788.79	
18	1	1	29741	1997.33	1692.32	1306.39	1110.28	880.91	666.55	
19	1	1	33309	1685.65	1791.84	1224.07	1390.34	1526.38	778.31	
20	1	1	34365	1031.05	1827.7758763	671.22343571	664.38	1000.0772301	343.82334721	
21	1	1	36602	1823.64	2119.2102202	1462.66	1672.98	2065.8686508	1821.89	
22	1	1	37699	2458.56	2279.23	2005.87	1064.63	1277.15	1028.07	
23	1	1	37866	2766.4	2252.53	2401.92	1900.94	1326.7	1834.43	
24	1	1	38218	1832.86	1302.79	1672.61	957.71	843.39	846.74	
25	1	1	38720	2173.26	915.55	1690.0244758	1107.67	721.24	1207.9461168	
26	1	1	39698	1423.32	2054.4533017	1662.0783782	728.55	866.90146059	621.74043735	
27	1	1	41559	1179.81	1644.27	1247.19	1240.21	1925.8	1280.98	
28	1	1	42594	1002.6	1165.39	725.4319289	1048.71	1086.84	642.90818993	
29	1	1	44872	1566.6	1847.86	1560.62	1225.72	1530.53	1188.49	
30	1	1	44941	1514.63	1440.0286922	1141.1255284	742.24	1215.3898184	1320.384614	
31	1	1	45137	1380.2	1328.34	1577.55	1268.85	1209.98	1619.8	
32	1	1	48311	1595.9	1725.11	1205.27	1412.62	928.2	868.36	
33	1	1	48525	1490.29	1150.12	1139.09	1079.79	1016.98	950.58	
34	1	1	48852	1803.07	3207.65	2460.57	1071.47	2111.85	1642.57	
35	1	1	49177	4086.36	1057.15	1629.210136	1836.3	627.68	1000.9169373	
36	1	1	49658	2033.09	2126.67	1336.24	889.28	837.45	442.64	
37	1	1	50868	2230.85	1757.82	1364.58	1198.7	1122.43	893.84	
38	1	1	54639	2621.0769432	1916.1176885	1420.83	904.48926142	787.376272	600.57	
39	1	1	54919	847.73	1339.21	1094.7	591.91	1131.31	920.23	
40	1	1	57209	883.4	1155.27	818.03	599.8	857.79	381.86	
41	1	1	57297	1310.82	1348.2694612	1246.2934717	754.55	1041.0236902	750.91295378	
42	1	1	57334	2061.32	1987.31	1971.66	1294.56	1321.71	1296.05	
43	1	1	58995	2474.5151674	3422.55	1164.3	1707.6474892	1982.67	873.33	
44	1	1	60701	2148.43	2280.9475387	1519.0983555	1088.51	1339.4350233	905.13685788	
45	1	1	60873	2204.8	1613.06	1767.48	1390.82	1191.61	1371.75	
46	1	1	64571	1379.58	1564.7	1237.46	986.79	1060.46	882.59	
47	1	1	67316	1514.15	1693.76	1726.37	1075.7	1370.1	1321.34	
48	1	1	67797	1229.21	1394.6408132	1174.8648019	803.29	647.85872496	992.39139533	
49	1	1	68732	900.95	1942.19	2100.0057203	667.69	1683.81	1838.0265729	
50	1	1	68840	876.78	551.94668885	624.34382986	497.43	233.49896841	360.80069168	
51	1	1	69497	1419.4	1639.5604769	1534.9700748	943.8	1503.7932195	1638.7681977	
52	1	1	71595	1206.6	1655.8506882	1007.110756	944.36	1395.3927548	1246.5049243	
53	1	1	71849	2822.16	2008.81	1806.22	1558.36	1182.46	1120.46	
54	1	1	72189	1013.77	1044.8850191	1010.1652712	500.51	1300.8504997	1287.6010567	
55	1	1	75626	1291.87	1324.8	1025.5	959.19	1262.65	802.55	
56	1	1	77954	1556.47	1735.99	1899.6876257	1313.29	1711.26	1582.5146529	
57	1	1	80407	1856.62	2429.5688534	1538.996741	1172.75	1012.8830134	565.02504116	
58	1	1	81061	3397.28	2802.8028037	2532.8827303	2107.52	1670.4658065	1520.1838841	
59	1	1	81364	1477.67	1682.56	1695.6354877	672.77	842.17	968.3092295	
60	1	1	81677	2366.85	1022.98	1539.7821315	1389.01	600.22	1314.5590705	
61	1	1	82876	1113.91	1208.74	1180.49	795.74	752.8	936.54	
62	1	1	87770	1331.18	1923.35	1890.06	936.41	2382.25	2035.07	
63	1	1	89740	2254.82	2324.73	1504.62	1218.12	1254.12	869.59	
64	1	1	91557	2169.46	1735.99	1694.89	1230.13	1047.89	1171.45	
65	1	1	93977	2246.75	1596.3766256	2304.1003448	1382.71	1330.2751971	1201.0734254	
66	1	1	94309	1145.69	1100.12	1458.84	628.18	941.48	1068.89	
67	1	1	95364	1324.91	1736.02	1547.72	1269.76	1916.11	1521.47	
68	1	1	95767	2317.43	1583.62	923.68	1104.73	882.74	503.2	
69	1	1	95986	948.07	1118.2	601.37301437	529.42	704.89	817.73843021	
70	1	1	97505	1833.25	1679.74	1832.57	1366.49	1533.45	1562.32	
71	1	1	98294	1438.1557879	2152.3895237	749.08	1132.1824651	1043.9986357	520.84	
72	1	1	98459	3871.1	1327.87	2173.86	1076.96	730.74	1457.28	
73	1	1	98644	2518.21	1968.25	2390.45	1459.65	1368.73	1529.19	
74	1	1	99888	2352.54	2838.34	2900.39	1305.81	1559.11	1606.95	
