# Supplementary material for: High Dietary Phosphorus Is Associated with Increased Breast Cancer Risk in a U.S. Cohort of Middle-Aged Women
Source: Nutrients. 2023 Aug 25;15(17):3735. doi: 10.3390/nu15173735 (PMC10490459; doi:10.3390/nu15173735)
Supplement: Supplementary file 1 [file nutrients-15-03735-s001.zip › Multiple Imputed Random Controls.rtf]

Model Information	
Data Set	WORK.IMPORT1	
Method	FCS	
Number of Imputations	25	
Number of Burn-in Iterations	20	
Seed for random number generator	713403001	


FCS Model Specification	
Method	Imputed Variables	
Regression	DTTKCAL0 DTTKCAL5 DTTKCAL9 DTTPHOS0 DTTPHOS5 DTTPHOS9	


Missing Data Patterns	
Group	DTTKCAL0	DTTKCAL5	DTTKCAL9	DTTPHOS0	DTTPHOS5	DTTPHOS9	Freq	Percent	
									
1	X	X	X	X	X	X	145	48.99	
2	X	X	X	X	X	.	1	0.34	
3	X	X	.	X	X	X	1	0.34	
4	X	X	.	X	X	.	47	15.88	
5	X	X	.	X	.	.	1	0.34	
6	X	.	X	X	.	X	20	6.76	
7	X	.	.	X	.	.	78	26.35	
8	.	X	X	.	X	X	1	0.34	
9	.	X	.	.	X	.	1	0.34	
10	.	.	X	.	.	X	1	0.34	

Missing Data Patterns	
Group	Group Means	
	DTTKCAL0	DTTKCAL5	DTTKCAL9	DTTPHOS0	DTTPHOS5	DTTPHOS9	
1	1852.346552	1691.673310	1618.920897	1125.526414	1114.629379	1125.243517	
2	3338.990000	3351.170000	2171.700000	1480.140000	1405.730000	.	
3	1559.670000	1956.480000	.	1004.050000	1284.150000	589.880000	
4	1740.593404	1810.472979	.	1140.624043	1228.607872	.	
5	1470.060000	874.110000	.	912.600000	.	.	
6	1905.866500	.	1872.876000	1160.119500	.	1345.289500	
7	1922.831026	.	.	1128.243846	.	.	
8	.	2692.500000	2078.880000	.	1552.970000	1417.920000	
9	.	1522.100000	.	.	945.730000	.	
10	.	.	2071.560000	.	.	666.290000	


Variance Information (25 Imputations)	
Variable	Variance	DF	Relative
Increase
in Variance	Fraction
Missing
Information	Relative
Efficiency	
	Between	Within	Total					
DTTKCAL0	12.712613	1487.348283	1500.569401	290.17	0.008889	0.008817	0.999647	
DTTKCAL5	332.345633	1468.403394	1814.042852	174.56	0.235385	0.192973	0.992340	
DTTKCAL9	977.485467	1192.422187	2209.007072	66.022	0.852538	0.469481	0.981567	
DTTPHOS0	4.591595	651.762934	656.538193	290.7	0.007327	0.007278	0.999709	
DTTPHOS5	127.539668	694.708518	827.349773	194.73	0.190931	0.162113	0.993557	
DTTPHOS9	579.991524	775.231239	1378.422425	71.19	0.778079	0.446360	0.982459	


Parameter Estimates (25 Imputations)	
Variable	Mean	Std Error	95% Confidence Limits	DF	Minimum	Maximum	Mu0	
DTTKCAL0	1861.750002	38.737184	1785.509	1937.991	290.17	1854.635540	1870.640796	0	
DTTKCAL5	1758.614718	42.591582	1674.554	1842.675	174.56	1715.819760	1796.663554	0	
DTTKCAL9	1674.035410	47.000075	1580.197	1767.874	66.022	1610.210621	1724.607591	0	
DTTPHOS0	1130.962088	25.623001	1080.532	1181.392	290.7	1126.470102	1136.839327	0	
DTTPHOS5	1151.272392	28.763688	1094.544	1208.001	194.73	1121.455772	1172.625572	0	
DTTPHOS9	1155.024168	37.127112	1080.998	1229.050	71.19	1116.386162	1197.711113	0	

Parameter Estimates (25 Imputations)	
Variable	t for H0:
Mean=Mu0	Pr > |t|	
DTTKCAL0	48.06	<.0001	
DTTKCAL5	41.29	<.0001	
DTTKCAL9	35.62	<.0001	
DTTPHOS0	44.14	<.0001	
DTTPHOS5	40.03	<.0001	
DTTPHOS9	31.11	<.0001	

Obs	_Imputation_	BRSTCAN	SWANID	DTTKCAL0	DTTKCAL5	DTTKCAL9	DTTPHOS0	DTTPHOS5	DTTPHOS9	
1	1	0	10629	3533.24	2566.26	2933.91	1657.17	1286.89	1945.29	
2	1	0	10801	1171.3	1517.28	1062.5150783	626.56	1065.32	677.53953859	
3	1	0	10910	2306.01	967.18	1594.94	1814.68	574.72	1085.74	
4	1	0	11180	3900.13	3378.05	2214.0787873	2387.1	1940.98	1451.983168	
5	1	0	11338	2306.28	1616.65	1187.31	1216.26	940.44	495.94	
6	1	0	11481	1842.58	1352.32	1179.87	1561.08	1254.44	1201.73	
7	1	0	11600	2145.04	1561.99	2434.47	1118.61	1007.77	1645.32	
8	1	0	11788	1310.48	1428.29	1307.42	612.42	529.7	814.75	
9	1	0	12183	1077.66	1335.88	829.61354983	658.96	1309.95	1329.530305	
10	1	0	12830	1912.08	1590.6	1726.33	1062.48	1272.3	1007.25	
11	1	0	12907	2149.5	2217.5045977	2164.6867692	1078.53	1259.4647306	1239.890286	
12	1	0	13621	1503.24	2267.7855888	1290.6	638.04	458.05566219	634.66	
13	1	0	13956	1127.32	1192.8	951.44	784.65	761.89	852.08	
14	1	0	14334	1803.775277	2692.5	2078.88	1118.3191032	1552.97	1417.92	
15	1	0	14596	1749.89	1164.09	1342.3346998	838.59	795.02	1457.6680194	
16	1	0	15150	1721.91	1683.26	1450.67	1242.95	1703.11	1195.44	
17	1	0	15210	1438.53	1466.3522284	841.22	655.17	562.94704561	408.21	
18	1	0	15976	1181.24	1469.67	1038.26	761.76	1123.58	763.95	
19	1	0	16115	1459.84	1149.8348235	1166.345783	784.88	867.24012791	691.23334123	
20	1	0	16214	1121.92	509.09	950.53	777.55	419.88	882.84	
21	1	0	16365	2767.56	2936.56	2623.32	1929.76	1909.1	1754.83	
22	1	0	16743	1543.85	1117.4	1086.69	745.42	569.86	648.57	
23	1	0	17288	1901.75	2131.4762978	1641.2509678	1330.55	1574.4790357	906.4435213	
24	1	0	17487	1354.7	1176.29	1187.28	797.85	556.54	641.15	
25	1	0	17851	681.85	3111.06	1651.4806005	617.16	2150.5	1148.6073964	
26	1	0	18151	1972.25	1935.7330603	2154.8828768	930	998.04235871	990.18879904	
27	1	0	18165	1902.37	2230.27	1727.1424804	1537.63	1727.84	1219.5919634	
28	1	0	18189	1620.97	2167.99	2036.767564	1041.44	1280.62	1331.1612764	
29	1	0	18414	1313.51	1252.55	1247.76	1233.58	131.55	1515.46	
30	1	0	19474	1475.52	1152.2	1444.05	1400.18	1387.29	1799.73	
31	1	0	19650	2121.02	1960.99	1585.97	1385.56	1082.82	837.36	
32	1	0	20047	2514.6	1629.51	1044.2588193	1581.72	1022.03	1041.019457	
33	1	0	20663	919.13	1502.55	650.06271189	720.83	1015.72	207.06054897	
34	1	0	21532	2427.24	1389.56	1821.59	1661.26	1095.71	1570.23	
35	1	0	21760	2163.26	1845.61	1577.08	2040.45	1660.33	1922.46	
36	1	0	21778	964.97	2399.4042829	2167.0656052	845.04	1155.1028121	507.4522849	
37	1	0	22142	1793.5	2290.72	2372.79	1306.88	2035.2	1529.3	
38	1	0	22364	2245.71	1164.2404672	1718.1680953	921.12	756.95206827	965.14428925	
39	1	0	22534	1936.04	1309.36	1679.52	1126	806.94	991.4	
40	1	0	22724	2461	2080.03	2452.83	1616.3	1244.14	1648.32	
41	1	0	23186	1272.27	1889.79	1194.92	428.95	726.62	544.15	
42	1	0	23205	1693.3	990.49173555	929.58416249	1213.36	745.84994609	825.91775167	
43	1	0	23445	2776.34	2646.5309805	3897.32	1210.25	1790.4613485	2068.83	
44	1	0	23459	2087.61	1759.29	1972.32	1226.96	1178.89	1172.39	
45	1	0	23805	2102.38	1596.4	1641.69	1450.04	1191.13	1219.11	
46	1	0	24170	888.8	1789.4265435	502.64981274	384.92	839.11298199	385.22319002	
47	1	0	24223	2490.84	2850.4272435	2490.84	1790.43	1458.7369659	1073.43	
48	1	0	24239	2355.3	1903.89	2048.85	1050.88	1085.6	1303.13	
49	1	0	24736	1308.15	2076.32	1138.85	636.88	1011.97	565.34	
50	1	0	25090	1093.05	2811.84	1544.25	610.95	1323.56	754.66	
51	1	0	25107	2053.37	1197.61	1125.92	850.55	629.35	930.8	
52	1	0	25389	1726.35	1658.44	1918.2277788	1215.21	1692.13	1566.8238399	
53	1	0	25959	1141.51	1206.24	1115.03	674.22	941.81	607.93	
54	1	0	26109	1106.75	841.42	672.76	606.83	389.53	601.3	
55	1	0	26305	1866.32	1510.7952649	1736.4594283	1597.83	1269.5296412	1336.5458359	
56	1	0	26812	1435.23	1735.4898245	1934.0408673	648.1	1024.1823033	1148.4132017	
57	1	0	27070	1516.7	1800.55	2443.7858789	1351.47	1363.09	1576.8009332	
58	1	0	27442	1747.25	1341.5686629	1692.0745533	635.92	908.0669714	1321.8220404	
59	1	0	27455	920.29	1175.64	1071.64	503.24	583.42	573.86	
60	1	0	27552	1914.39	1241.71	1177.32	944.55	975.92	749.8	
61	1	0	28147	2705.39	1641	2383.01	1616.51	1102.03	1944.98	
62	1	0	28341	1268.33	1029.14	892.04	684.5	566.69	486.35	
63	1	0	28616	1330.56	1412.77	1334.21	742.98	655.63	674.14	
64	1	0	28778	2414.91	1984.81	1729.24	1169.79	814.13	759.57	
65	1	0	28895	1272.86	2182.7982716	1921.3	754.55	1081.8483556	1388.28	
66	1	0	29059	3403.06	1307.7891733	2345.4446094	1659.89	1258.3754286	2097.7786248	
67	1	0	29084	1588.88	1433.47	1795.6923194	1126.5	1121.8	1520.1103757	
68	1	0	30395	1237.36	1169.1510333	711.45591057	792.2	358.29035845	381.77742946	
69	1	0	31012	1879.2	2098.0983366	3092.18	1557.91	1529.7243287	2393.91	
70	1	0	31237	1595.28	1597.17	1288.2115507	1206.39	1229.22	925.47519867	
71	1	0	31546	2009.85	1037.43	1435.02	1008.39	450.01	636.45	
72	1	0	31557	1480.65	1276.54	914.51	883.69	817.9	654.86	
73	1	0	31837	3713.31	1353.96	1656.49	3356.88	1163.07	1304.13	
74	1	0	32118	1914.97	1481.12	1446.95	1133.48	1345.37	1721.02	
75	1	0	32476	2167.98	1159.3088647	2095.6392218	1442.59	513.02201512	1507.7837749	
76	1	0	33185	882.82	1061.41	1177.85	715.43	798.18	927.42	
77	1	0	33276	2457.1	2671.62	2592.56	1377.77	1452.16	1481.16	
78	1	0	33521	4057.09	2030.1850895	2310.7599747	1525.21	1186.4666443	1613.7417707	
79	1	0	33533	1762.57	1312.02	932.15	1006.48	772.51	583.72	
80	1	0	33890	1982.66	1539.84	1063.51	1132.88	782.82	753.36	
81	1	0	34146	1578.99	1136.37	1545.79	865.66	543.98	647.21	
82	1	0	34183	1635.45	1621.4846984	1975.0321283	986.32	1040.2160073	1031.2474038	
83	1	0	34659	2371.71	2741.69	2838.6923991	1475.59	1615.23	1976.8186952	
84	1	0	34677	1047.71	1723.9037043	1412.5066523	798.84	802.58285445	797.98323442	
85	1	0	35326	1594.5	1194.48	1841.75	1167.32	1136.09	2106.58	
86	1	0	35328	1119.57	1032.51	-324.5575223	784.06	604.28	14.54102817	
87	1	0	35613	1831.12	1063.4824047	1201.3573824	865.66	784.55586115	760.09345171	
88	1	0	35770	1543.86	2341.47	1947.63	944.32	1408.45	1206.67	
89	1	0	35795	2406.37	1320.7875721	1674.6038941	1190.74	1088.3733059	1344.511599	
90	1	0	35955	933.52	1342.36	1258.31	532.83	686.96	575.83	
91	1	0	36018	646.35269762	1522.1	1227.5243151	20.792722699	945.73	9.4979481208	
92	1	0	36311	1604.97	1100.61	847.27	878.54	629.58	556.59	
93	1	0	36766	2722.6	1883.0624475	1468.8208515	2144.95	1435.3210996	1577.1883821	
94	1	0	37296	2979.88	664.24	1990.9495972	1885.78	622.54	2152.8304795	
95	1	0	37373	1958.77	2113.1614691	2123.0295634	1349.91	1479.9607113	1067.4758277	
96	1	0	37479	1846.16	2599.01	2439.65	1081.76	1782.91	1626.12	
97	1	0	37599	2026.39	1325.89	1608.876376	1069.38	995.98	1113.590407	
98	1	0	37947	1984.26	1473.21	1134.73	1503.33	1311.35	773.91	
99	1	0	38138	1926.62	1503.4493343	2079.4809176	1028.73	1253.9805044	1822.8052011	
100	1	0	38786	1533.19	1044.14	1999.8825309	1071.77	981.29	1771.3912118	
101	1	0	39056	2228.71	2634.81	3118.2409754	1348.97	1897.21	2034.4144702	
102	1	0	39278	1752.15	1389.63	1817.31	1264.54	1055.23	1408.46	
103	1	0	39528	2085.1	1951.4003567	1931.7085345	1437.37	1060.1857292	1986.4483922	
104	1	0	39819	1721.76	1720.8	1918.18	1043.09	1517.52	1541.21	
105	1	0	40044	2410.27	2536.6738966	1866.8160219	1234.07	1684.3341388	1172.3317497	
106	1	0	40131	1276.81	1035.67	1153.03	660.33	874.88	846.34	
107	1	0	40877	2268.62	1829.2907464	2756.2878577	1370.93	2107.9123654	2370.0695002	
108	1	0	41581	1362.12	2817.82	1628.8916381	817.25	1550.01	784.59439729	
109	1	0	41657	1849.63	1404.92	1446	1076.44	637.3	1719.4	
110	1	0	42150	2237.32	1910.3977286	2370.78	1557.09	1502.3147361	1726.84	
111	1	0	42362	1434.87	1149.17	1263.59	1083.63	1183.19	1048	
112	1	0	42521	1310.29	2478.8941306	2066.03	658.02	864.29201844	1212.32	
113	1	0	42713	2120.05	1948.61	1956.75	1586.2	1767.92	1495.95	
114	1	0	42891	2383.14	2068.2041054	2345.2303841	1626.15	1111.4754819	1484.3175392	
115	1	0	42976	2519.09	2018.5114388	2573.8510619	1372.91	1267.18438	1443.9630239	
116	1	0	43170	1349.96	1303.0927794	1275.33	630.11	814.9751424	605.25	
117	1	0	43324	2244.17	3958.24	3596.73	1383.72	1818.44	1451.69	
118	1	0	43505	2137.94	2026.65	1557.73	1088.73	1348.13	863.64	
119	1	0	43572	3461.64	1197.7635662	2708.3961429	1846.42	1229.1164045	1663.8730233	
120	1	0	43722	2323.55	1705.979594	2784.97	1314.8	1352.0177746	1823.89	
121	1	0	43792	1058.49	2376.4817469	1211.4686014	761.43	1502.7442138	968.14243401	
122	1	0	44036	2158.13	2317.23	2323.63	1821.95	1856.79	2075.84	
123	1	0	44230	1431.8	1604.71	1777.5320966	1539.15	1288.3	1995.2764123	
124	1	0	44395	1648.66	1144.8	1326.53	1208.61	1187.02	904.46	
125	1	0	45072	1564.92	1978.53	1501.69	790.4	1068.02	714.01	
126	1	0	45196	3044.94	2160.7751819	3077.9573779	1888.38	1442.9878857	1823.264793	
127	1	0	45210	3058.71	1938.3379235	2738.03	2400.57	1941.3584991	3516.43	
128	1	0	45689	1666.56	1862.8315733	2303.79	1175.54	1474.8726126	1999.35	
129	1	0	45746	2916.33	2547.0572282	2194.8142834	1588.73	1507.7181306	1194.4777256	
130	1	0	46094	1732.65	1731.2769495	1253.7371187	1157.07	984.11503839	978.30426333	
131	1	0	46254	2354.84	1909.9853776	1627.4289603	1081.39	1523.6987755	1519.3066897	
132	1	0	47423	1416.05	1323.39	1519.07	677.81	640.96	848.54	
133	1	0	47595	1238.14	3148.94	2008.6645647	1141.38	1926.43	1356.3896463	
134	1	0	47789	2815.54	1775.27	1671.86	1401.79	979.57	1217.6	
135	1	0	47805	1387.85	1438.54	1529.2352623	664.84	713.57	1404.6113079	
136	1	0	47844	1436.17	1278.73	1272.13	1154.35	912.21	749.15	
137	1	0	48041	1132.92	1058.8261449	1663.6813864	621.6	304.06957887	795.68021388	
138	1	0	48104	2398.48	1793.74	1803.7	1517.27	1371.95	1546.15	
139	1	0	48491	2179.04	2113.07	1758.99	1094.39	1471.4	1090.77	
140	1	0	48515	1658.99	1898.31	2010.80885	1088.93	1858.92	1044.8027132	
141	1	0	48532	2566.76	3330.9	2646.1	1496.34	1915.79	1856.5	
142	1	0	49252	2228.75	2201.7835397	2378.709655	2128.42	1650.3239513	2007.8692374	
143	1	0	49618	1190.69	1823.9911234	1352.184128	639.76	826.86340986	228.36315786	
144	1	0	49770	2350.11	1502.46	1665.79	1072.33	963.89	1688.67	
145	1	0	49850	2732.74	2437.1	1748.78	1498.19	1363.71	1578.17	
146	1	0	50183	1781.53	1772.9598134	1579.5345332	1050.24	1543.2679939	1422.1110148	
147	1	0	50997	1277.87	1566.41	845.83	702.83	1329.78	733.79	
148	1	0	51582	1670.23	1032.1137299	1014.5340589	991.16	917.70749512	1003.2016949	
149	1	0	51641	2307.49	2245.47	1702.1518161	1461.21	1277.65	1046.2284636	
150	1	0	51878	2221.39	1572.3	2413.58	1687.45	1082.55	2425.66	
151	1	0	52487	1574.86	1461.6861598	1400.4331855	794.19	851.59474621	1285.0835205	
152	1	0	52503	1654.53	1616.22	1027.86	839.79	766.62	487.32	
153	1	0	53185	2024.71	2832.91	2836.09	1161.25	2200.95	1518.84	
154	1	0	53287	1205.6	657.28070598	780.00176619	850.88	464.23603823	633.12808828	
155	1	0	53438	2115.65	2253.1003749	2119.0027335	1399.53	662.68476007	1035.3703326	
156	1	0	53669	1900.12	2476.26	1838.6	709.29	1375.43	999.62	
157	1	0	53899	1551.38	1135.27	2627.06	970.49	736.85	1531.68	
158	1	0	53945	1394.21	1601.24	1208.8659954	653.89	585.44	295.73631441	
159	1	0	54054	1325.92	1376.0207843	1226.3069881	955.27	495.29768298	753.01293926	
160	1	0	54996	1835.5	1551.48	1495.62	1396.08	693.53	665.24	
161	1	0	56433	969.91	710.09167168	969.91	819.97	744.84760577	960.79	
162	1	0	56821	1921.58	1412.72	1367.61	1309.08	1239.64	915.53	
163	1	0	56838	1247.01	1690.7	1704.56	1462.14	2080.67	1660.35	
164	1	0	56880	2620.3	2713.06	3208.74	1114.16	1429.5	1982.56	
165	1	0	58648	3338.99	3351.17	2171.7	1480.14	1405.73	1242.9769897	
166	1	0	58837	2344.76	2421.11	1202.67	1501.8	1897.69	980.16	
167	1	0	58906	2131.85	1641.01	1522.4	1395.74	1170.95	1084.28	
168	1	0	59427	1500.16	1849.5370353	1322.0233217	804.43	883.63612073	792.07101447	
169	1	0	59740	774.35	610.87	933.32	565.37	595.13	1110.46	
170	1	0	60495	1978.46	1579.9	2212.1494704	1546.05	1081.63	1928.849227	
171	1	0	60504	1841.21	1008.16	1390.03	681.17	497.87	714.06	
172	1	0	60981	1308.73	1436.33	2245.3205483	860.09	902.87	1626.4597589	
173	1	0	61564	1900.51	1406.34	1055.4	901.09	761.08	612.85	
174	1	0	62189	1509.07	1491.37	1456.93	1052.02	1358.51	968.06	
175	1	0	62806	1694.17	1700.53	1453.64	782.34	1084.57	1013.22	
176	1	0	63342	1454.63	1398.58	1313.38	1282.23	914	910.95	
177	1	0	63783	887.57	2159.34	710.89471574	425.07	1088.68	455.88638805	
178	1	0	64716	1847.73	1989.9989425	1054.4817645	869.61	1283.1165446	1301.4021626	
179	1	0	64754	1244.86	225.30252842	741.06886578	974.53	596.65572904	913.60514379	
180	1	0	64794	3468.03	2155.14	2005.12	2048.32	1677.37	1474.56	
181	1	0	65261	1335.32	1551.8741571	2421.9934813	1023.92	1418.0256149	1263.0165647	
182	1	0	65941	1843.29	2322.59	2201.62	1790.85	2280.62	1978.69	
183	1	0	66169	1231.11	1606.6309041	1898.5099077	646.83	1251.1056496	1325.3629247	
184	1	0	66677	2754.89	1916.107124	1778.5519257	2267.68	1701.3761155	1691.1302264	
185	1	0	66739	2773.5	1967.0490754	1926.778394	1462.49	1873.8776651	1467.2855273	
186	1	0	67010	843.96	1728.0455734	743.9996102	737.6	1065.6788464	284.4577882	
187	1	0	67011	1900.8	822.15592413	963.11130409	950.5	639.60728607	211.53991683	
188	1	0	67248	971.99	74.240824408	438.23834766	698.55	33.128640867	545.87610783	
189	1	0	67305	971.34	2448.9401843	2008.9631769	666.19	1672.7930023	1045.1435632	
190	1	0	67432	1759.34	1921.39	1801.5	1076.47	1287.5	1501.16	
191	1	0	67827	1759.06	2532.4476135	2244.6956027	736.14	1083.3083012	762.42655409	
192	1	0	67961	1998.59	1542.04	1858.1364175	1100.17	1012.78	1203.141878	
193	1	0	67996	1056.17	2869.8559651	2660.5932756	581.7	1829.9773062	1982.9468769	
194	1	0	68894	1826.45	1397.1716316	1792.8697578	1363.22	634.19313879	923.99603137	
195	1	0	69343	1228.65	784.99	1290.9551048	712.8	621.4	1125.5803487	
196	1	0	69503	2591.48	1182.2281231	1288.08	1034.02	767.07228698	1490.42	
197	1	0	69649	1999.99	1625.76	1646.25	1213.95	1073.81	1248.32	
198	1	0	69838	2737.46	2077.8292759	2781.8307621	1681.18	1503.4169703	2598.5195126	
199	1	0	69969	1897.69	1416.4744149	2461.9789664	1237.88	912.46022028	1782.0383989	
200	1	0	70970	2028.64	1272.23	2137.06	1224.3	857.26	1378.25	
201	1	0	71067	1078.84	946.97	1031.22	822.33	728.23	714.41	
202	1	0	71305	1981.92	3189.62	1984.28	1169.52	1403.34	1028.28	
203	1	0	71464	1932.92	2229.6843099	1710.8746973	1199.47	1386.4452902	1380.2095126	
204	1	0	72165	1940.01	1807.01	1313.17	1029.46	863.15	1208.42	
205	1	0	72369	1524.03	1808.57	1492.27	580.3	807.11	728.03	
206	1	0	72576	3592.93	2837.24	2085.37	1255.54	962.97	1249.19	
207	1	0	72702	1884.95	1734.21	1795.22	982.35	1018.66	1340.31	
208	1	0	72933	1923.69	1854.04	1542.06	1173.25	1349.09	1178.08	
209	1	0	74581	1781.83	1824.98	1276.9	1042.88	709.03	876.57	
210	1	0	74786	3151.92	2590.43	2623.7929794	1768.53	1411.34	1780.2802052	
211	1	0	74861	3259.83	1177.098281	1030.89	2198.03	1197.8187521	1041.73	
212	1	0	75503	1134.15	2240.6785249	1284.1120226	573.9	1021.0531019	795.38787458	
213	1	0	75529	1641.86	2417.52	1462.06	1031.54	1666.46	1092.06	
214	1	0	75543	2413.48	1520.11	2163	864.6	714.75	1365.55	
215	1	0	75761	2965.67	2019.4717258	2758.2494659	1778.54	1531.6533288	1964.4648648	
216	1	0	76366	1500.52	1181.25	1372.62	727.43	576.51	710.55	
217	1	0	76649	1698.15	1351.6217984	1962.2059	1294.19	1469.1447855	1933.5708329	
218	1	0	76743	2749.83	2364.3	1985.92	2379.44	2372.4	1770.74	
219	1	0	77385	1454.41	1122.08	1938.8860585	695.83	890.88	1397.4784589	
220	1	0	77394	1448.98	946.65	1695.74	1020.72	770.58	1149.31	
221	1	0	77776	2162.81	1906.0876708	2635.5077778	745.28	634.97137607	1166.4341117	
222	1	0	77809	1759.33	1141.3935969	1450.1220085	987.32	1007.9114944	1461.6358849	
223	1	0	78016	1296.63	1074.91	1444.89	828.55	538.1	971.08	
224	1	0	78068	3851.75	2311.5908209	2466.2504229	2272.28	1549.1652068	2014.1697945	
225	1	0	78420	1199.9	1981.98	1049.4279617	1369.6	1657.62	1082.6351404	
226	1	0	78451	1322	1043.68	420.52224809	807.69	729.72	692.43698855	
227	1	0	78455	1408.23	1022.1	1035.81	964.04	709.72	805.67	
228	1	0	78546	2055.74	2219.05	2272.51	846.73	949.88	1155.9	
229	1	0	79043	1202.47	2174.52	2135.1710331	1041.41	1507.07	1202.0394943	
230	1	0	79727	875.11	2244.5609765	1577.07	543.12	1657.4758494	864.71	
231	1	0	79954	1441.92	1928.95	1577.01	1248.6	1305.36	1058.69	
232	1	0	80157	2614.84	2799.83	2269.7297429	2155.75	2041.88	2127.0304176	
233	1	0	80653	1995.98	1520.52	2002.45	924.59	953.86	1168	
234	1	0	80655	1104.36	1793.51	1530.7109768	705.15	1054.38	1110.5320382	
235	1	0	80761	1715.92	1568.6888545	1673.3354223	671.81	1041.4579659	1417.9374489	
236	1	0	80893	1733.62	1237.69	2021.07	1181.15	848.36	1257.09	
237	1	0	81100	3222.07	2530.9	1717.21	1257.81	1323.2	847.88	
238	1	0	81430	2083.34	2646.1860662	2516.1728504	1276.2	1378.9300596	1726.5583137	
239	1	0	81837	2130.46	1914.0718742	2387.5562719	1529.93	1519.3460774	1909.4164184	
240	1	0	82166	1357.22	1874.07	1143.37	644.75	905.71	610.33	
241	1	0	82255	1686.75	1668.24	1463.38	845.53	960.78	804.59	
242	1	0	82605	2173.31	1589.73	1602.79	1384.49	1112.1	1101.82	
243	1	0	82609	1943.01	1981.5700588	1519.1272884	992.84	1375.8502567	898.01455884	
244	1	0	82905	1716.86	1351.16	1247.37	2318.33	1756.41	1204.87	
245	1	0	83011	2145.76	1556.86	2019.2271339	1314.56	1299.57	1758.5557955	
246	1	0	83354	973.72	1338.15	1088.49	567.8	930.63	747.8	
247	1	0	83433	1698.61	1757.28	1289.72	861.78	824.39	645.05	
248	1	0	83953	1227.51	1587.44	2189.3083097	703.77	950.44	1534.3485368	
249	1	0	84331	2324.26	2604.95	3314.06	936.31	1414.47	1678.91	
250	1	0	84525	1644.36	1548.2247597	1263.26	740.61	803.83915064	705.65	
251	1	0	84646	1559.26	1804.9	1671.78	861.51	946.52	1169.45	
252	1	0	84992	3281.78	3149.91	2376.16	1838.14	2652.59	1795.8	
253	1	0	85286	912.69	1734.1197987	2232.4590952	762.57	945.58913673	1204.591907	
254	1	0	85300	2802.2	2222.73	1510.74	1566.66	1614.84	899.82	
255	1	0	85314	1401.72	1136.82	1447.1641882	771.04	780.83	1015.7301982	
256	1	0	85446	1491.31	1775.32	1255.93	926.52	1374.37	892.59	
257	1	0	85485	1970.34	1624.5487784	1670.06	1179.64	1069.0994015	1305.13	
258	1	0	85658	1001.86	1330.0535741	1691.8929699	435.31	1286.3769781	1117.5254859	
259	1	0	87042	4735.06	3937.08	1979.29	2359.08	1889.33	1254.79	
260	1	0	87166	1971.9601043	2398.0763702	2071.56	883.17832664	1284.9064731	666.29	
261	1	0	87205	1559.67	1956.48	955.48398844	1004.05	1284.15	589.88	
262	1	0	87737	1713.03	1417.19	1619.11	843.85	855.91	896.93	
263	1	0	88194	2119.69	1676.3978119	1371.0615332	827.26	641.35966558	545.35342044	
264	1	0	88284	1263.81	1536.62	1776.43	724.85	636.2	909.49	
265	1	0	88436	2899.88	852.36	2107.475542	1341.81	668.13	2093.9306793	
266	1	0	88448	924.61	1344.86	1240.17	601.93	2039.52	1278.68	
267	1	0	89112	2026.84	1503.44	1734.8492432	1455.87	1100.64	1530.9766655	
268	1	0	89364	1949.93	1769.3792378	1791.862425	1182.44	705.18030108	1397.8367869	
269	1	0	89467	1659.5	2023.79	1545.8	913.17	911.67	727.29	
270	1	0	89456	1728.13	1578.31	2222.72	969.48	988.25	1849.96	
271	1	0	89581	2030.21	2497.0734181	2340.1478982	1513.56	1663.2737413	1656.9968629	
272	1	0	90243	1142.86	1186.94	1044.35	560.01	822.83	756.31	
273	1	0	90794	1203.11	1602.06	1538.65	622.63	1191.34	1248.72	
274	1	0	91574	2352.17	1718.88	2191.4862638	1237.55	1121.45	1481.4966613	
275	1	0	91580	1145.11	1426.94	993.44	622.69	1045.33	864.55	
276	1	0	91710	1470.06	874.11	1640.2681958	912.6	834.46559092	1492.5683472	
277	1	0	92464	972.06	2737.18	2469.11	743.26	1669.58	1532.8	
278	1	0	92723	1658.16	1863.14	1957.1823128	984.77	1097.66	1495.8949771	
279	1	0	93492	1580.04	765.74	729.97	975.89	517.69	643.23	
280	1	0	93615	1950.32	2088.79	1297.22	1580.05	1526.02	932.39	
281	1	0	93727	1795.54	1408.57	1493.63	1724.26	1384.67	1219.73	
282	1	0	93998	1449.93	1688.9055808	1028.1685707	887.44	962.84159241	435.57129977	
283	1	0	94159	2170.49	1358.89	1351.95	937.11	1606.31	804.48	
284	1	0	94589	1521.49	2304.22	2379.4604613	1037.26	1623.68	1459.81206	
285	1	0	94937	1688.08	1498.57	1595.04	1225.99	778.18	1330.11	
286	1	0	95398	1852.2	1647.9734133	774.27	1107.91	514.97888107	358.85	
287	1	0	95756	1759.53	921.54	1394.93	835.83	711.23	941.57	
288	1	0	95757	2117.3	2835.82	2155.1959152	1662.8	1470.85	1231.2658126	
289	1	0	95760	1277.3	1590.06	1590.45	1222.95	1304.32	1700.95	
290	1	0	96121	971.57	1130.4485595	1041.0421823	721.08	602.3292019	233.18874769	
291	1	0	96187	4064.45	2742.7757581	2907.4860846	1741.02	1419.3542072	1806.4134699	
292	1	0	96478	1352.06	1935.91	1483.21	1004.75	1273.42	994.07	
293	1	0	97723	2651.46	809.67	2717.8	1608.95	833.5	1538.13	
294	1	0	97995	2270.18	2378.0257461	2394.7120633	956.94	1235.6234451	1206.0126595	
295	1	0	98030	1646.7	2266.248681	1811.59	1236.61	1510.3234374	1327.11	
296	1	0	98106	1157.85	710.87	882.98	614.19	634.77	777.45	
